# The Short-Chain Fatty Acid Butyrate Attenuates Pulmonary Vascular Remodeling and Inflammation in Hypoxia-Induced Pulmonary Hypertension

**DOI:** 10.3390/ijms22189916

**Published:** 2021-09-14

**Authors:** Vijaya Karoor, Derek Strassheim, Timothy Sullivan, Alexander Verin, Nagavedi S. Umapathy, Edward C. Dempsey, Daniel N. Frank, Kurt R. Stenmark, Evgenia Gerasimovskaya

**Affiliations:** 1 Department of Medicine Cardiovascular and Pulmonary Research Laboratory, University of Colorado Denver, Denver, CO 80204, USA; vijaya.karoor@cuanschutz.edu (V.K.); derek.strassheim@cuanschutz.edu (D.S.); timothy.sullivan@cuanschutz.edu (T.S.); edward.dempsey@cuanschutz.edu (E.C.D.); kurt.stenmark@cuanschutz.edu (K.R.S.); 2 Vascular Biology Center, Augusta University, Augusta, GA 30912, USA; AVERIN@augusta.edu (A.V.); USIDDARAMAPPA@augusta.edu (N.S.U.); 3 Center for Blood Disorders, Augusta University, Augusta, GA 30912, USA; 4 Rocky Mountain Regional VA Center, Aurora, CO 80045, USA; 5 Division of Infectious Diseases, Department of Medicine, University of Colorado Denver, Denver, CO 80204, USA; daniel.frank@cuanschutz.edu; 6 Division of Critical Care Medicine, Department of Pediatrics, University of Colorado Denver, Denver, CO 80204, USA

**Keywords:** butyrate, pulmonary hypertension, vascular remodeling, inflammation, vascular permeability, endothelial barrier, HDAC inhibitors, protein acetylation

## Abstract

Pulmonary hypertension (PH) is a progressive cardiovascular disorder in which local vascular inflammation leads to increased pulmonary vascular remodeling and ultimately to right heart failure. The HDAC inhibitor butyrate, a product of microbial fermentation, is protective in inflammatory intestinal diseases, but little is known regarding its effect on extraintestinal diseases, such as PH. In this study, we tested the hypothesis that butyrate is protective in a Sprague–Dawley (SD) rat model of hypoxic PH. Treatment with butyrate (220 mg/kg intake) prevented hypoxia-induced right ventricular hypertrophy (RVH), hypoxia-induced increases in right ventricular systolic pressure (RVSP), pulmonary vascular remodeling, and permeability. A reversal effect of butyrate (2200 mg/kg intake) was observed on elevated RVH. Butyrate treatment also increased the acetylation of histone H3, 25–34 kDa, and 34–50 kDa proteins in the total lung lysates of butyrate-treated animals. In addition, butyrate decreased hypoxia-induced accumulation of alveolar (mostly CD68+) and interstitial (CD68+ and CD163+) lung macrophages. Analysis of cytokine profiles in lung tissue lysates showed a hypoxia-induced upregulation of TIMP-1, CINC-1, and Fractalkine and downregulation of soluble ICAM (sICAM). The expression of Fractalkine and VEGFα, but not CINC-1, TIMP-1, and sICAM was downregulated by butyrate. In rat microvascular endothelial cells (RMVEC), butyrate (1 mM, 2 and 24 h) exhibited a protective effect against TNFα- and LPS-induced barrier disruption. Butyrate (1 mM, 24 h) also upregulated tight junctional proteins (occludin, cingulin, claudin-1) and increased the acetylation of histone H3 but not α-tubulin. These findings provide evidence of the protective effect of butyrate on hypoxic PH and suggest its potential use as a complementary treatment for PH and other cardiovascular diseases.

## 1. Introduction

Pulmonary hypertension (PH) is a progressive disease characterized by increased pulmonary vascular resistance and structural vascular remodeling, ultimately leading to right heart failure [1,2]. Inflammation in both the lung vascular and right ventricular (RV) compartments are major contributing factors to the pathology of PH [3]. In addition, studies from animal models and in patients with pulmonary arterial hypertension (PAH) have suggested a possible role of alterations in the gut microbiota in disease pathogenesis [4,5,6]. Emerging data provide a link between blood pressure regulation and microbiota-derived-metabolites, such as the short-chain fatty acid butyrate, which is also known to possess HDAC inhibitory activity [4,7,8]. To date, different treatment strategies have been used for PH, including endothelin-1 (ET1) receptor antagonists, prostacyclin analogs, cGMP- phosphodiesterase (PDE) inhibitors, and Ca^2+^ channel blockers, as well as some anti-inflammatory and metabolic agents, which variably provide relief of symptoms [9,10,11]. However, these strategies do not cure the disease, indicating that the molecular mechanisms involved in the pathogenesis of PH are not completely understood.

An epigenetic regulation of gene expression has been implicated in the pathogenesis of PH [12,13]. Histone and non-histone protein acetylation is regulated via the activation of acetyltransferases (HAT) and deacetylases (HDAC) and is fundamental to disease-associated changes in vascular and cardiac cell phenotypes [12,14]. HDAC inhibitors, commonly known as a class of anti-cancer agents [14], have been recently found to have therapeutic benefits in PH and other cardiovascular diseases [15,16]. Alterations in the activities of HDACs and their expression has been observed in cells and tissues in preclinical models of PH and human PH. For instance, the expression levels of HDAC1 and HDAC5 were found to be higher in lungs from both PH patients and experimental hypertensive animals [15]. In pre-clinical models, HDAC inhibitors suppress hypoxia-induced PH by exerting anti-proliferative and anti-inflammatory effects in vascular cells [15,17]. In addition, class I HDAC inhibitors markedly decreased mRNA expression levels of several cytokine/chemokine (including IL-6, CCL2, CXCL12, CCL5, and GM-CSF) in pulmonary adventitial fibroblasts from chronically hypoxic hypertensive calves, further supporting a beneficial therapeutic effect of HDAC inhibitors in PH [18].

Butyrate, a four-carbon short-chain fatty acid, is known as an endogenous HDACi derived from the microbial fermentation of dietary fiber [19]. Butyrate inhibits class I and II HDAC (but not HDAC class III) and is involved in a widespread epigenetic regulation [20,21]. HDAC inhibition is an important mechanism of butyrate action, in addition to its signaling via G protein coupled-receptors (GPR41, GPR43, and GPR109), and cellular uptake, leading to metabolic utilization in cellular energy pathways [20]. Several studies have demonstrated the beneficial effects of butyrate in the treatment of diseases with a pathogenic inflammatory component, such as metabolic syndrome, ischemic stroke [22], atherosclerosis [23], inflammatory bowel disease [19,24], renal fibrosis [25], and acute lung injury [26]. In vitro studies demonstrated a protective effect of butyrate against hypoxia-induced angiogenesis [27] and endothelial barrier dysfunction [28,29]. The therapeutic effects of butyrate are due to its anti-proliferative, anti-inflammatory, cytoprotective, differentiating, and metabolic properties [20,30,31,32]. Studies on smooth muscle cells (SMC) demonstrated that the anti-proliferative effects of butyrate are mediated by the regulated expression of proteins involved in cell cycle progression and by the inhibition of Akt signaling [33,34]. In fibroblasts, butyrate inhibits proliferation and decreases proline-reach protein synthesis, hyaluronate, and collagen [35,36]. The effects of butyrate in endothelial cells are linked to NO production [37], the downregulation of endothelin-1 expression [38], and inhibition of angiogenesis via the attenuation of HIF-1α, VEGF-, and COX-mediated signaling [27,39,40]. The anti-inflammatory effects of butyrate are mediated via decreased inflammatory cytokine production and down-regulation of VCAM-1 and ICAM-1 in endothelial cells [29,41,42].

Our previous studies on rodent models of PH demonstrated the accumulation of inflammatory cell infiltrates in the lung and around large vessels, suggesting that pulmonary vascular inflammation and remodeling could be associated with hypoxia-induced endothelial barrier dysfunction [43,44]. However, in contrast to the well-studied barrier-protective effect of butyrate in epithelial cells in intestinal inflammatory diseases, its effect on vascular endothelial cells remains unexplored. In this study, using the SD rat model of hypoxic PH, we examined the beneficial effect of butyrate on multiple pathological outcomes of PH, including RVSP, RV hypertrophy, pulmonary vascular remodeling, permeability, cytokine production, protein acetylation, and macrophage accumulation in the lung. Studies on rat microvascular endothelial cells (RMVEC) provided evidence of barrier-protective effects of butyrate and showed butyrate-mediated upregulation of tight junctional proteins, increased acetylation of subsets of cellular proteins, including histone H3 and α-tubulin. Together, our study suggests a protective effect of butyrate in hypoxic PH and possibly other cardiovascular diseases.

## 2. Results

### 2.1. Butyrate Prevented RVSP and RV Heart Hypertrophy in Hypertensive Hypoxic SD Rats

**Figure 1:**
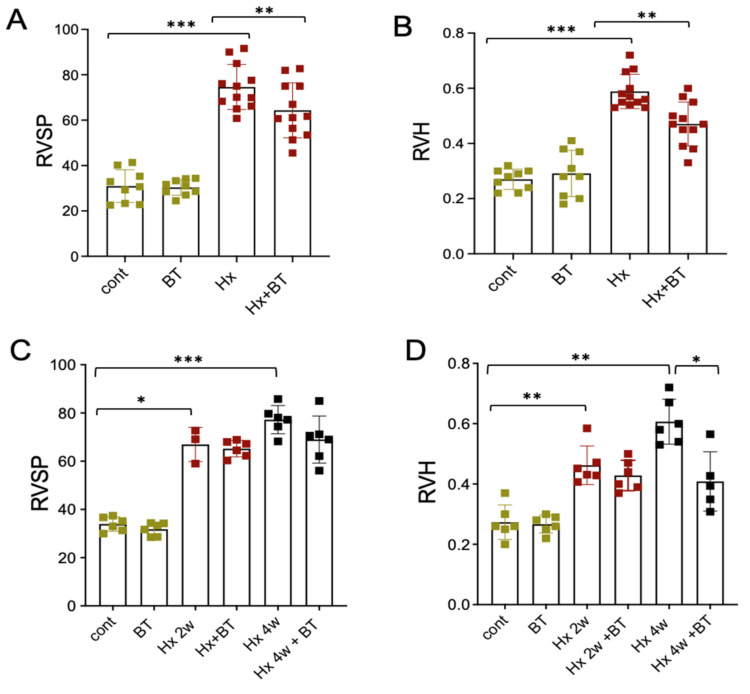
Effects of butyrate treatment on RVSP and RV heart hypertrophy in hypertensive hypoxic SD rats. (**A**,**B**): Animals were subjected to hypoxic conditions (Hx) or remained at Denver altitude (cont) as for 4 weeks described in the “Materials and Methods”. Butyrate (BT) was given in drinking water at the concentration of 220 mg/kg. Hemodynamic studies and evaluation of RV hypertrophy (Fulton index, RV/LV + S) were performed at the end of the experiments (cont and BT groups (n = 9), Hx and Hx + BT groups (n = 12)); (**C**,**D**): Animals were subjected to control (cont) or hypoxic (Hx) conditions for 2 and 4 weeks. Part of the animals from these groups were used for RVSP and RV hypertrophy measurement. The other part received butyrate treatment (BT, Hx + BT) for additional 2 weeks, (cont and BT groups (n = 6), Hx (2 weeks n = 3, 4 weeks n = 6), and Hx + BT groups (n = 6)). Data represent a mean +/− SED; * *p* < 0.05, ** *p* < 0.01, *** *p* < 0.001.

SD rats exposed to hypobaric hypoxia (4 weeks; 18,000 feet elevation) demonstrated a significant increase in right ventricular systolic pressure (RVSP) and right ventricular (RV) hypertrophy (heart Fulton index) (Figure 1A,B). Supplementation with butyrate in drinking water (220 mg/kg intake) attenuated the development of hypoxia-induced RVSP and RV hypertrophy (Fulton index; RV/LV+S) in hypertensive hypoxic animals. In reversal experiments, butyrate treatment (220 mg/kg), when it was given after two (Figure 1C) and four weeks of hypoxic exposure (not shown), did not significantly reverse elevated RVSP and RV hypertrophy. When butyrate (2200 mg/kg) was given after four weeks of hypoxic exposure, it significantly reversed RV hypertrophy but had an insignificant effect on elevated RVSP.

### 2.2. Butyrate Attenuated Hypoxia-Induced Pulmonary Vascular Remodeling

**Figure 2:**
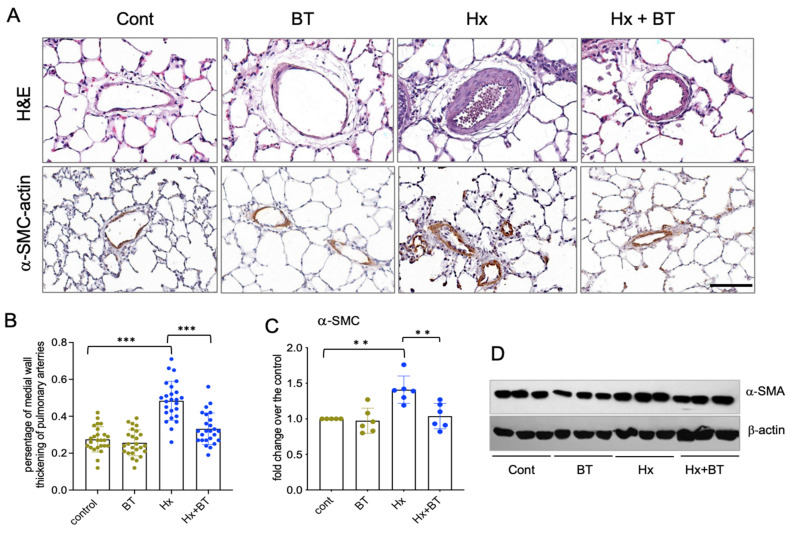
Butyrate attenuates hypoxia-induced pulmonary vascular remodeling. (**A**): Lung sections from control (cont), butyrate-treated (BT), hypoxic (Hx), and butyrate-treated hypoxic (Hx + BT) animals were subjected to H&E and immunohistochemical analyses with anti α-SMC antibodies; scale = 100 µM; (**B**): Analysis of medial wall thickening of distal pulmonary arteries. Scanned images of pulmonary arteries (n = 25 for each group) were evaluated using Image Scope software as described in “Materials and Methods”. Data represent a mean +/− SED; *** *p* < 0.001; (**C**,**D**): Western blot analysis of α-SMA expression in total lung lysates. Results are representative from six animals (n = 6) obtained from two independent experiments; data represent a mean +/− SED; ** *p* < 0.01.

Pulmonary vascular remodeling is an important pathophysiological component of PH. To investigate the effect of butyrate on pulmonary vessels, we performed histological and IHC analyses of lung tissue sections. H&E staining and quantitative evaluation of pulmonary vascular remodeling demonstrated that hypoxia significantly increased the medial thickening of distal arteries (diameter 60–100 µm) that was markedly attenuated by butyrate (Figure 2A,B). In addition, the elevated expression of α-SMA was observed in the distal pulmonary vessels of the hypoxic animals, and this response was markedly attenuated by butyrate (Figure 2A,C,D).

### 2.3. Butyrate Attenuated Hypoxia-Induced Accumulation of Inflammatory Cells in the Lung

**Figure 3:**
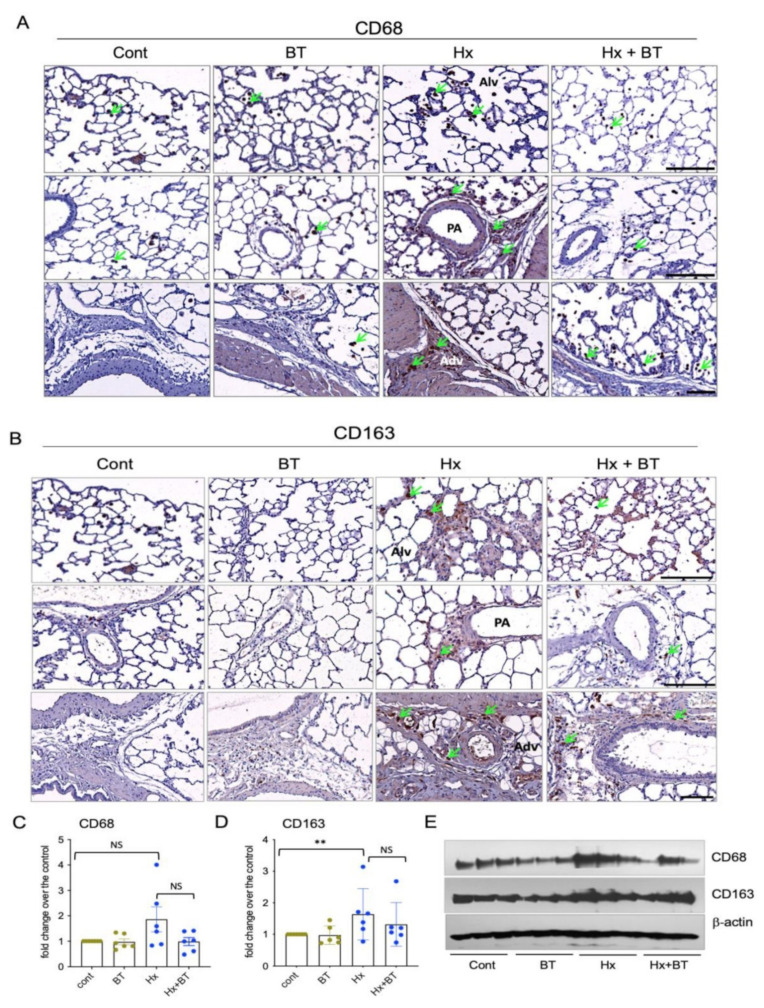
Hypoxia and butyrate modulate the level of macrophage accumulation in the lungs. (**A**,**B**): Lung sections from control (cont), butyrate-treated (BT), hypoxic (Hx), and butyrate-treated hypoxic (Hx + BT) animals were subjected to immunohistochemical analyses with anti-CD68 and anti-CD163. (The upper row on both panels shows lung alveoli (Alv), middle row shows distal pulmonary vessels (PA = pulmonary artery), and the lower row shows vascular adeventitia (Adv) and surrounding perivascular space), arrows indicate CD68+ and CD163+ macrophages; scale = 200 µm on upper and middle panels, scale = 100 µm on lower panels; (**C**–**E**): Western blot analysis of CD68 and CD163 expression in total lung lysates. Results are representative from six animals (n = 6) obtained from two independent experiments; data represent a mean +/− SED; ** *p* < 0.01; NS = not significant.

Pulmonary macrophages are critical contributors to pulmonary vascular inflammation and remodeling [45]. To assess the anti-inflammatory effects of butyrate, we performed an immunostaining analysis of lung sections for the expression of CD68 (pan-macrophage) and CD163 (M2 type macrophage) markers. Figure 3A shows that some amounts of CD68+ macrophages are present in the lungs at the baseline level in both control (untreated) and butyrate-treated rats. However, the lungs of hypoxic animals demonstrated increased accumulation of CD68+ macrophages, especially in the perivascular areas. Butyrate treatment of hypoxic rats partially attenuated the accumulation of CD68+ cells in the lungs, yet some CD68+ cell patches remained in the perivascular and peribronchial areas as well as the CD163+ perivascular areas. Butyrate treatment of hypoxic rats partially attenuated the accumulation of CD68+ cells in the lungs, yet some CD68+ cell patches remained in the perivascular areas. Furthermore, the CD163+ macrophages were almost undetectable in the lungs of control and butyrate-treated rats, with only a few cells detected in the perivascular space (Figure 3B). In contrast, hypoxic lung specimens demonstrated a robust accumulation of CD163+ macrophages in the alveolar spaces and perivascular areas of middle- and large-size vessels. Butyrate treatment significantly attenuated the accumulation of CD163+ macrophages, yet some patches of CD163+ cells remained present in the perivascular areas. The Western blot analysis of total lung lysates showed an upregulation of CD68 and CD163 expression in hypoxic lungs (yet it did not reach statistical significance for CD68) and showed a dynamic toward a partial decrease in CD163+ and CD68+ cell accumulation in response to butyrate treatment of hypoxic animals (Figure 3C–E).

### 2.4. Butyrate Exhibits Endothelial Barrier Protective Effect In Vivo and In Vitro

**Figure 4:**
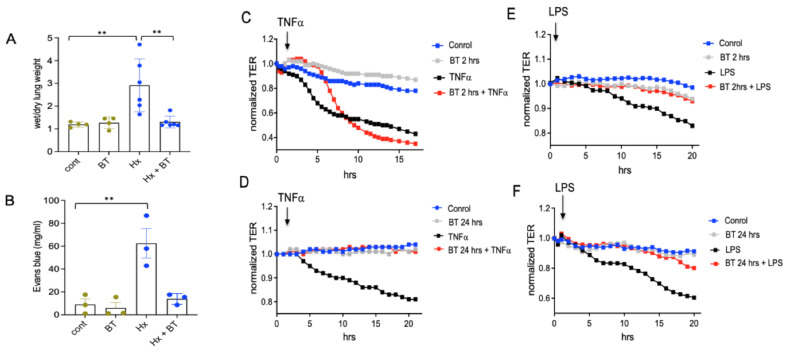
Butyrate exhibits endothelial barrier protective effect in vivo and in vitro. (**A**,**B**): Evaluation of pulmonary edema and vascular leak. An EBD solution (30 mg/mL) was infused by tail vein injection at a dose of 30 mg/kg for 10 min. Right and left lungs were isolated and processed as described in “Materials and Methods”. Shown are the data of one representative experiment from three; data represent a mean +/− SED; ** *p* < 0.01; (**C**–**F**): Transendothelial electrical resistance (TER) assay in cultured rat microvascular endothelial cells (RMVEC). Cells were grown in ECIS array units (Electric Cell Impedance Sensing System), as described in “Materials and Methods”, pretreated with butyrate for 2 or 24 h, and stimulated with or without TNFα (40 ng/ml) or LPS (1 µg/ml). Each panel shows one representative experiment from three.

To examine if hypoxia-induced vascular inflammation was associated with vascular leakage and can be prevented by butyrate, we performed wet and dry lung weight measurements and an Evans Blue dye (EBD) permeability assay. We found that butyrate effectively prevented hypoxia-induced pulmonary vascular edema and vascular leak in hypoxic SD rats (Figure 4A,B). To clarify the role of butyrate in regulating endothelial permeability in vitro, RMVEC, grown to confluence in ECIS chambers, were pretreated with butyrate (1 mM) for either 2 or 24 h before an edemagenic agonist (LPS and TNFα) challenge. Transendothelial electrical resistance (TER, an inverse index of permeability) was monitored for 20 h. As shown in Figure 4C,D, while pretreatment with butyrate for 2 h transiently attenuated the drop in TER, induced by TNFα (with a full inhibition of the response for up to 8 h of TNFα treatment), the prolonged pretreatment with butyrate (24 h) had a more permanent effect (with the inhibition of the maximal response at 20 h of TNFα treatment by 80%). In the case of LPS (Figure 4D,E), either 2 or 24 h pretreatments are almost completely abolished LPS-induced permeability increase (with the inhibition of the maximal responses at 20 h of LPS treatment by 89% and 75%, respectively). Overall, these data support the protective role of butyrate in endothelial barrier compromise.

### 2.5. Butyrate Upregulates the Expression of Tight Junctions in Lung Microvascular Endothelial Cells

**Figure 5:**
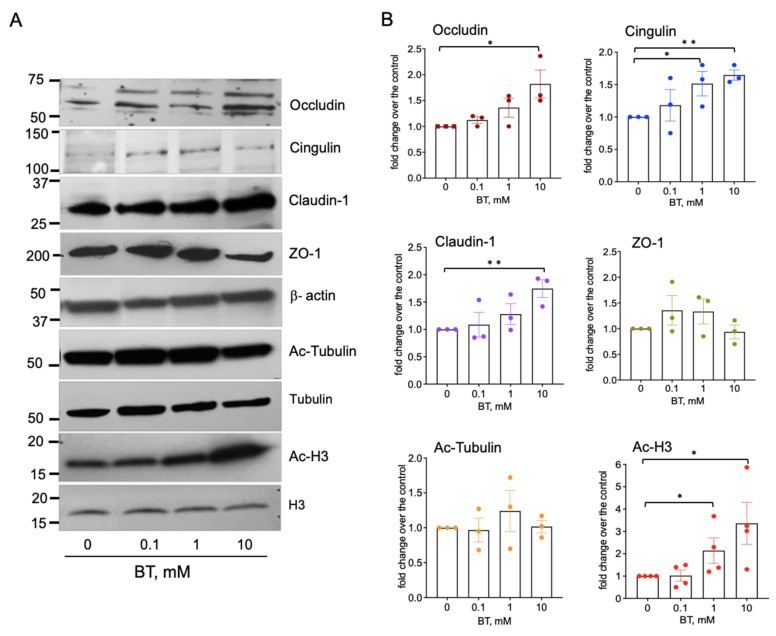
Butyrate upregulates the expression of tight junctional proteins in rat lung microvascular endothelial cells. (**A**): Western blot analysis of tight junctional proteins, Ac-tubulin, and Ac-H3 in RMVEC. Growth-arrested cells (48 h, DMEM without serum) remained untreated or stimulated with butyrate (0.1, 1, and 10 mM) for 24 h. Total cell lysates (20–30 μg) were subjected to Western blot analysis (**B**): Densitometric analysis of Western blot data show the relative intensity of protein expression in butyrate-stimulated cell versus without treatment; data represent a mean +/− SED from three to four independent experiments; * *p* < 0.05; ** *p* < 0.01.

Tight junctions are critically important to endothelial barrier properties; therefore, we determined if butyrate regulates the expression of tight junctional proteins. The Western blot analysis of RMVEC incubated with butyrate (0–10 mM, for 24 h) revealed a concentration-dependent upregulation of occludin, claudin-1, and the microtubule-associated protein, cingulin (Figure 5). An insignificant increase in the expression of junctional protein ZO-1 was also observed. In addition, butyrate treatment increased the acetylation of histone H3 (Acetyl-H3) but not α-tubulin, indicating that butyrate may regulate endothelial barrier function, which is likely via epigenetic mechanisms.

### 2.6. Butyrate Increases Protein Acetylation in the Lung

**Figure 6:**
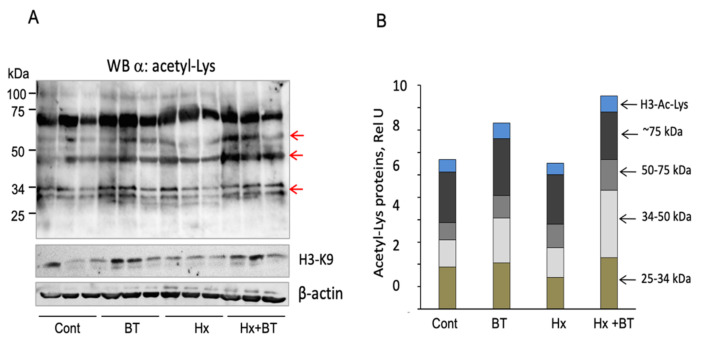
Butyrate increases protein acetylation in the lungs. (**A**): Total lung extracts (30 μg) obtained from each individual animal were analyzed by Western blot with anti-Ac-Lys and anti-Ac-H-Lys (K9) antibodies. Arrows indicate proteins that showed increased acetylation levels and H3-Ac-Lys; (**B**): Quantitative analysis of protein acetylation. Arrows indicate protein ranges (25–34 kDa, 34–50 kDa, 50–75 kDa, and ≈75 kDa) used for quantitative analysis; shown are data from three independent experiments.

Butyrate intake (220 mg/kg) with drinking water increased butyrate concentration in the serum (of both the control and hypoxic animals) from 3.75–3.50 μM to ≈44.0 μM. To determine if this butyrate treatment affects protein acetylation in the lungs, we performed Western blot analysis of total lung lysates with anti-Ac-Lys and anti-Acetyl-Lys-H3 antibodies. As shown in Figure 6A,B, butyrate increased the acetylation level of proteins in the range of 25–34 kDa and 34–50 kD and increased the level of Ac-H3-K9, suggesting butyrate’s HDAC inhibitory activity toward both the histone and non-histone proteins.

### 2.7. Hypoxia and Butyrate Regulate Cytokine Production in the Lung

**Figure 7:**
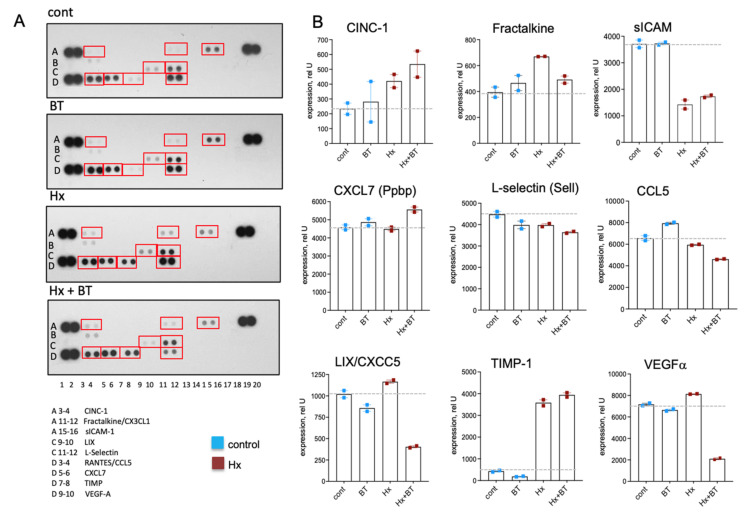
Butyrate modulates cytokine levels in the lungs. Cytokine levels were measured in total lung lysates using a Proteome Profiler^TM^ Array. An equal amount of protein from six animals of the same experimental condition was pooled, and 400 μg of pooled lysate were used for the assay and loaded in duplicates; data represent a mean +/− SED; (**A**): Cytokine array panels showing the various cytokines in rat lung extracts; (**B**): Densitometric analysis of cytokine expression.

Chemokines and cytokines play a critical role in the inflammatory responses contributing to the pathogenesis of PH [46,47,48] and can be regulated via HDAC inhibitory mechanism [49]. Using a cytokine array panel, we observed the increased expression of CINC-1, Fractalkine, and TIMP in the lungs of hypoxic animals compared to the controls (Figure 7A,B), whereas the level of soluble ICAM (sICAM) was decreased. Butyrate treatment decreased the expression of LIX, VEGF, and to a lesser extent, Fractalkine and CCL5 in hypoxic lungs. In hypoxic animals, butyrate slightly stimulated CINC-1 and CXCL7. Notably, a high expression level was observed for CCL5, VEGFα, CXCL7, L-selectin, and TIMP compared to other cytokines, indicating the physiological importance of these cytokines for hypoxia-associated inflammatory responses.

## 3. Discussion

PH remains an incurable disease, for which the pathogenic mechanisms remain unclear. Emerging evidence indicates a role of histone and non-histone protein acetylation in PH, implying epigenetic modulation as a therapeutic approach for PH. The microbiota-derived HDAC inhibitor butyrate can be absorbed into the circulation from the gastrointestinal tract and exhibit physiological effects in many tissues, suggesting that butyrate and other gut microbial metabolites could be critical contributors to cardiovascular and lung diseases [8,19,24,50]. Considering previous findings on the protective effect of HDAC inhibitors and butyrate in cardiovascular diseases, in this study, we investigated if butyrate supplementation would be beneficial in hypoxic PH.

Our findings demonstrate that butyrate prevented the hypoxia-induced increase in RVSP and RV hypertrophy when it was given during the course of hypoxic exposure. In the “reversal” experiments, butyrate effectively decreased elevated RVH but not RVSP in four-week hypoxic animals. The inability of butyrate to completely prevent and reverse the PH symptoms can be explained by epigenetic mechanisms underlying PH pathogenesis perhaps involving multiple HDAC isoforms as well as other mechanisms, including methylation; whereas butyrate selectively inhibits class I and class II HDACs [20,21]. Noteworthy, butyrate has a more potent inhibitory effect on hypoxia-induced RVH compared to RVSP, which may suggest a higher expression of butyrate-sensitive HDAC isoforms in the heart tissue compared to the lungs. In addition, butyrate was more potent to prevent than to reverse PH symptoms. This observation is consistent with previous research showing that disease regression takes more time than development because structural changes in the heart the lungs are hardly reversible [51,52].

Hypoxia-induced pulmonary vascular remodeling is a distinctive feature of pulmonary hypertension. Histological examination of structural lung changes by H&E and IHC staining for α-SMA expression in lung sections from control, hypoxia, and butyrate-treated rats revealed significant vascular wall medial thickening and the muscularization of distal pulmonary vessels in hypertensive animals. These structural changes and the expression of SMC α-actin were prevented by butyrate treatment. Similar to our observations, studies on the MCT model of PH demonstrated protective effects of the butyrate derivative, 4-phenyl butyric acid, on PH-associated vascular remodeling through the attenuation of endoplasmic reticulum stress in the lungs and SMCs [53,54].

Inflammation has been recognized as an important pathophysiological component of pulmonary hypertension and correlates with the level of pulmonary vascular remodeling. Both the alveolar and interstitial macrophages play an important role in lung inflammation [45,55,56]. We observed increased levels of CD68+ (a common macrophage marker) and CD163+ (M2 macrophage marker) cells in the lungs. The CD68+ cells were significantly accumulated in perivascular alveolar space close to large vessels and the adventitia of the vascular wall, which is consistent with the previous observations showing that, at least in part, CD68+ macrophages may represent vascular infiltrates. In turn, CD163+ macrophages have been found in the fibrotic areas of the alveolar space, in perivascular space, around the vessels, and in the vascular adventitia. Butyrate treatment reduced both CD68+ and CD163+ macrophages in the lungs. Similar to our findings, increased interstitial and perivascular monocyte/macrophage accumulation was shown in MCT and hypoxic models of PH [57,58], as well as in lung sections of patients with idiopathic PAH [3]. The accumulation of macrophages and other inflammatory cells, including T cells, B cells, and dendritic cells, was found in the pulmonary perivascular space and around the plexiform and other lung lesions, confirming the crucial role that immune and inflammatory responses play in the pathogenesis of idiopathic PAH [56]. Regarding macrophage phenotypes, data in the mouse model of PH suggest that at early hypoxic exposure, alveolar and perivascular/interstitial macrophages exhibit similar pro-inflammatory phenotypes, whereas on day 14, interstitial macrophages exhibit phenotypic reprogramming toward an anti-inflammation and pro-reparative state [59]. Consistent with these observations, we detected more CD163+ (compared to CD68+), macrophages in the lungs of chronically hypoxic rats. The mechanisms of butyrate-mediated inhibition of inflammatory responses in the lungs are not fully defined, but some data support a decrease in leukocyte adhesion to EC via downregulation of VCAM-1 and ICAM-1 expression by butyrate [29,60]. These results provide novel evidence of the anti-inflammatory effects of butyrate in the lungs, pointing out that HDAC inhibition can be considered as an anti-inflammatory therapy in PH.

Hypoxia and hypoxia-associated inflammation in the lungs are important contributors to pulmonary vascular leak in PH [46,61,62,63]. EBD extravasation assay demonstrated that butyrate attenuated hypoxia-induced vascular permeability in hypertensive animals. These observations along with a profound inhibitory effect of butyrate on VEGFα production suggest that VEGF may play a key role in the increased vascular permeability in hypoxic animals. In addition, the contribution of other cytokines and extracellular nucleotides, such as ATP, to pulmonary vascular permeability is very likely, as ATP and other nucleotides can be increased in the extracellular space under hypoxic conditions [64,65,66]. The barrier-protective effect of butyrate observed in the lungs was reproduced in vitro on RMVEC. In the TER assay, we showed a barrier-protective effect of butyrate against TNFα- and LPS-mediated endothelial barrier disruption. These data are in agreement with the barrier-protective effects of HDAC6 inhibitor tubacin A, which prevented endothelial barrier dysfunction in response to thrombin [67], TNFα, and LPS [68] in endothelial cells and the mice model of acute lung injury (ALI). In both hypoxic PH and ALI, the protective effect of butyrate on LPS-mediated barrier dysfunction may have physiological importance, as elevated LPS in the lungs and circulation may result from gut dysbiosis and the overgrowth of Gram-negative bacteria. Together, our data provide evidence on the protective effects of butyrate against vascular inflammation, endothelial activation, and vascular remodeling in PH.

While the barrier protective and anti-inflammatory effects of butyrate have been investigated in colonic epithelial cells in the relation to gastrointestinal diseases [24,69], less is known regarding the mechanisms of butyrate action in vascular endothelial cells. Butyrate and other HDAC inhibitors increase epithelial barrier integrity via the upregulation of junctional proteins [70,71], which suggests a link between epithelial barrier function, cellular differentiation, and morphogenesis [72,73]. Our data on RMVEC showed butyrate-induced upregulation of tight junctional proteins ZO-1, occluding, and claudin-1, as well as a cytoplasmic adaptor protein, cingulin, which has been previously reported to improve endothelial barrier function in vitro and in vivo [74]. In HUVEC, acetate, butyrate, and propionate, and HDAC inhibitor, trichostatin, have been shown to decrease tight junctional permeability, providing additional evidence for a barrier-protective effect of butyrate in endothelial cells [28]. The upregulation of junctional proteins in endothelial cells by butyrate may also suggest its effect on endothelial phenotypic differentiation, which would promote vascular stabilization and anti-angiogenesis in hypoxic conditions.

Our data demonstrated that taken with drinking water, butyrate exhibits “out-of-gut” effects and prevented a hypoxia-induced deacetylation of histone H3 and 25–34 kDa and 34–50 kDa proteins in the lungs. The observed protein profiles also indicate that butyrate regulates both histone and non-histone protein acetylation. However, a more detailed cell-specific analysis of protein acetylation, along with a comparative analysis of HDAC isoform expression and their inhibition by butyrate, would provide further insights into the molecular mechanism of butyrate actions in the lungs.

Increased levels of several cytokines have been observed in patients with PAH and contributed to pulmonary vascular remodeling by stimulating the accumulation of extracellular matrix production, contractility, and proliferation of SMC [55]. In addition to the regulation via post-translational modification, cytokines can also be regulated by the reversible acetylation and deacetylation of histones [49]. Considering the anti-inflammatory effect of butyrate, we examined its effects on cytokine production in the lungs. Analysis of cytokine profiles in lung tissue lysates showed a hypoxia-induced upregulation of TIMP-1, CINC-1, and Fractalkine and downregulation of soluble ICAM (sICAM). The expression of Fractalkine, VEGFα, but not CINC-1, TIMP-1, and sICAM was downregulated by butyrate. These cytokines play a role in cell growth, differentiation, angiogenesis, and inflammatory cell recruitment, thereby contributing to pulmonary vascular remodeling in PH [45,75,76,77].

As mentioned above, butyrate is a product of the microbial fermentation of dietary fiber [19]. Mounting evidence supports a link between alterations in the gut microbiota composition [78] and the development of cardiovascular diseases [5,7,79]. An increased Firmicutes/Bacteriodetes ratio (F/B) along with a reduced gut microbial diversity has been reported as a signature of intestinal dysbiosis in MCT and AngII models of PAH [5,7,79]. Supplementary to our main study, the observations on the SD model of PH showed that chronic hypoxia had minimal effects on overall microbiota composition, but nonetheless, multiple taxa differed in relative abundance between groups, showing a lesser abundance in hypoxic SD rats (Appendix sections Appendix A.1 and Appendix A.2). Genera belonging to the phyla Firmicutes are upregulated in chronically hypoxic animals, which is consistent with a previous report showing increased Firmicutes in hypertension [7,79]. However, the specific effects of chronic hypoxic exposure on intestinal microbiota composition as well as the identification of butyrogenic bacterial species remain to be a subject of further investigation, considering the importance of a “gut–lung” connection in hypertension [8,80]. In addition, some data provide evidence that exogenous butyrate may increase the abundance of butyrate-producing bacteria in different animal models, suggesting a restoration effect of butyrate on intestinal microbiome composition and homeostasis [81,82,83,84]. In this regard, the possibility should be explored as to whether butyrate can protect against hypoxia-induced imbalance in gut microbiota in hypoxic PH.

In conclusion, our study presented new evidence of the protective effects of the HDAC inhibitor butyrate on pathophysiological outcomes in experimental hypoxic PH. An important question remains regarding more detailed molecular mechanisms of butyrate actions in endothelial and other vascular and lung cells, which lead to the prevention of pathological vascular leak and hyper-proliferative vascular responses in hypoxic PH. The toxicity of HDAC inhibitors is a serious impediment even with subtype-selective inhibitors, which remains one of the more common reasons why any therapeutic regime for the treatment of PH is not fully successful [85]. The observed beneficial effects of naturally produced HDAC inhibitor butyrate may suggest its complementary use with traditional pharmacological drugs for the treatment of PH and possibly other cardiopulmonary diseases.

## 4. Materials and Methods

### 4.1. Experimental Animals

SD rats (Charles River Laboratory, Strain 400; eight weeks old) were placed in hypobaric chambers (50.6 kPa, simulated an average atmospheric pressure at 18,000 feet elevation) for 4 weeks, while control (normoxia) groups were kept at Denver altitude in temperature-controlled rooms (22–25 °C) with a 12:12 h light–dark cycle. All experiments were approved by the University of Colorado Animal Care and Use Committee. In the “Prevention” study, half of the animals from both the control and hypoxic groups were treated with potassium butyrate (PubChem, Bethesda, MD, USA) in drinking water (220 mg/kg and 2200 mg/kg intake). In the “Reversal” study, butyrate treatment of control and hypoxic groups was started after 2 or 4 weeks and continued for another 2 weeks. Untreated groups received tap water. At the end of the experiments, right ventricular systolic pressure (RVSP) and right ventricular hypertrophy (Fulton index, LV/RV+S) were determined as pathophysiological outcomes of PH. The concentration of butyrate in serum that was quantitated using ELISA (Aviva Systems Biology, San Diego, CA, USA) increased from 3.75 ± 0.50 and 3.50 ± 0.56 μM (in control and hypoxic animals, respectively) to 44.00 ± 1.63 and 44.00 ± 2.16 μM (in control and hypoxic animals, respectively) in response to butyrate treatment.

### 4.2. Hemodynamic Studies and Evaluation of Right Ventricular Hypertrophy

Rats were anesthetized using an isoflurane precision vaporizer (Smiths Medical, Minneapolis, MN, USA) with anesthetic induction at 5% isoflurane concentration and maintenance at 2.5%. Anesthetized rats were placed in a supine position, spontaneously breathing isoflurane/air mixture through a rodent nosecone. A 22-gauge 1-inch needle connected to a fluid-filled disposable Transpac^®^ IV transducer (Hospira, Lake Forest, IL, USA) was introduced under the xiphoid process of the animal, and the needle was advanced into the right ventricle. An RV pressure waveform was verified and recorded using an MP100 data acquisition system (BioPac Sytems, Inc., Goleta, CA, USA) and an Apple iMac computer running AcqKnowledge version 3.9.1 (BioPac Sytems, Inc., Goleta, CA, USA). Analyses were carried out on the acquired pressure waveforms using the AcqKnowledge software [86]. For the measurement of right ventricular (RV) hypertrophy, heart ventricles and septum were dissected, dried for 7 days, and weighed. Lungs were collected for histology, protein, and cytokine analyses.

### 4.3. Pulmonary Vascular Leak

Rats were lightly anesthetized using isoflurane gas, and Evan’s blue dye (EBD) solution (PubChem, Bethesda, MD, USA) was infused by tail vein injection at a dose of 30 mg/kg for 10 min. Then, rats were euthanized with an overdose of isoflurane, and the heart and lungs were flushed with saline. After weighing the right and left lungs separately, the right lung was frozen in liquid nitrogen for EBD extraction, while the left lung was allowed to dry until a stable weight was obtained for the calculation of wet-to-dry weight ratios. The EBD was extracted from lungs incubated for 24 h in formamide (100%, 60 °C). The extracted dye was quantified by measuring the absorption at 620 nm against standards of Evan’s blue dissolved in formamide and normalized to the calculated dry weight of tissue. Units are expressed as ng/mg of dry tissue.

### 4.4. Immunohistochemical Analysis

Lungs were fixed in 10% phosphate-buffered saline (PBS)–formalin for at least 24 h and embedded in paraffin. Immunohistochemical analysis of paraffin-embedded sections (4 μm) was performed using Vectastain Universal Quick Kit (Vector Laboratories Inc., Burlin, CA, USA) according to the manufacturer’s recommendations. Briefly, slides were washed in gradual dilutions of ethanol for deparaffinization. Antigen retrieval was achieved by incubating the slides in heated (100 °C) citrate buffer (pH = 6.0; Dako, Santa Clara, CA, USA). Endogenous peroxidase activity was suppressed by 3% hydrogen peroxide treatment. Then, slides were incubated with a blocking buffer (1% universal horse serum) for 1 h at room temperature, the primary antibody overnight at 4 °C, and with an HRP-labeled secondary antibody for 30 min at room temperature. Positive staining was detected using a 3,3′-diaminobenzidine substrate chromogen system (Dako), which was followed by counterstaining with hematoxylin. Primary antibodies used were CD68 (1:100, BioRad, Hercules, CA, USA), CD163 (1:100, Abcam, Cambridge, MA, USA), and acetyl-tubulin (1:100, Santa Cruz Biotechnology, Dallas, TX, USA).

### 4.5. Evaluation of Pulmonary Artery Medial Thickness

H&E-stained lung sections were scanned using a Leica Versa (Aperio, Sausalito, CA, USA) whole slide scanning microscope with 20× objective (Leica Biosystems, Buffalo Grove, IL, USA). The pulmonary arteries (n = 25 for each group, external diameter 60–100 µm) were randomly selected for high-power resolution and evaluated using Image Scope software. Pulmonary vascular remodeling was accessed by measuring the percentage of medial thickness, which was calculated as follows [87]: medial wall thickness (%) = (external diameter − internal diameter)/external diameter) × 100%.

### 4.6. Western Blot Analysis

Quick-frozen SD rat lungs were homogenized (PowerGen 700; Pittsburgh, PA, USA) in RIPA buffer containing protease inhibitors (Thermo Scientific Scientific, Waltham, MA, USA). After centrifugation (13,000× *g*, 20 min), supernatants (20–40 μg) were subjected to Western blot analysis with anti-α-SMA (1:1000, Sigma Aldrich St. Louis, MO, USA), anti-CD68 (1:1000, Bio-Rad, Hercules, CA, USA), anti-CD163 (1:1000, Abcam, Cambridge, MA, USA), anti-Ac-Tubulin (1:1000, Santa Cruz, Dallas, TX, USA), anti-Ac-lysine, or Ac-H3-K9 (1:1000, Cell Signaling Technology, Danvers, MA, USA) antibodies. To determine the effect of butyrate on the expression of junctional proteins, rat lung microvascular endothelial cells (RMVEC, CellBiologics, Chicago, IL, USA) were cultured in DMEM/10%FBS and growth-arrested in DMEM without serum for 48 h before stimulation with butyrate (0–10 mM, 24 h). Then, cells were lysed in RIPA buffer with protease inhibitors (Thermo Fisher Scientific) and centrifuged (12,000× *g*, 10 min). Supernatants (20–30 µg of total protein) were subjected to Western blot analysis with anti-occludin (1:1000, Invitrogen, Waltham, MA, USA), anti-cingulin (1:500, Santa Cruz Biotechnology), anti-ZO-1 (1:1000, Santa Cruz Biotechnology), anti-claudin-1 (1:1000, Cell Signaling Technology), anti-histone H3 K9 (1:1000, Cell Signaling Technology), anti-histone H3 (1:2000, Cell Signaling Technology), anti-Ac-tubulin (1:1000, Santa Cruz Biotechnology), anti-tubulin (1:1000, Cell Signaling Technology), and β-actin (1:1000, Sigma-Aldrich) antibodies. After incubation with HRP-conjugated secondary antibody (1:30,000 Azur Biosystems, Dublin, CA, USA), blots were developed via chemiluminescence and were quantified using ImageJ software (NIH). The β-actin was used as a loading control.

### 4.7. Transendothelial Electrical Resistance (TER)

RMVEC were grown in DMEM and supplemented with 10% FBS until confluence on gold microelectrodes coated with cysteine (10 mM) in ECIS array units (Electric Cell Impedance Sensing System; Applied Biophysics, Troy, NY, USA). Cells were pretreated with butyrate for either 2 or 24 h, which was followed by either LPS (1 µg/mL) or TNFα (40 ng/mL) stimulation. Transendothelial electrical resistance (TER) was measured as we have previously described [88].

### 4.8. Cytokine Analysis

Cytokine levels were measured in total lung lysates using a Proteome Profiler ^TM^ Array (rat cytokine array panel; R&D Inc, Minneapolis, MN, USA) according to the manufacturer’s instructions. Equal amounts of protein (100 μg) from six animals of the same experimental group were pooled into one sample. For each experimental condition, 400 μg of pooled lysate was used for the cytokine array. Cytokine levels were quantified by densitometric analysis of the array panels using ImageJ software.

### 4.9. Statistical Analysis

Quantitative data are expressed as the mean ± SEM. Statistical analyses were performed using GraphPad Prism 4.0 software. Student’s unpaired *t*-test followed by a Mann–Whitney nonparametric post-test were used to assess the statistical differences between the groups of data. *p* < 0.05 was considered statistically significant.

## Figures and Tables

**Figure A1:**
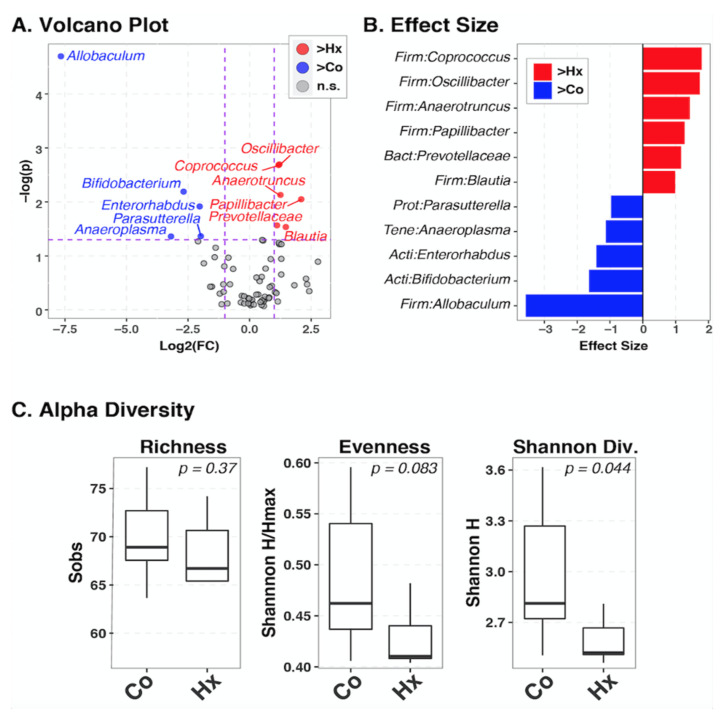
Identification of bacterial taxa differing between hypoxia and control groups. Between-group differences in the relative abundance of individual bacterial taxa were identified using the ANOVA-like differential expression (ALDEx2) test, which takes into account the compositional nature of microbiota datasets. Taxa with fold-changes >2 and nominal *p*-values <0.05 (Welch test) are considered significant; (**A**): plot of log2(fold-change) vs. −log10(*p*-values). Taxa in the upper right quadrant, colored red, were significantly enriched in the hypoxia group (Hx) compared to controls (Co). Conversely, taxa in the upper left quadrant, colored blue, were significantly enriched in the control group compared to hypoxia. Gray circles in bottom quadrant were not significant (n.s.). Vertical and horizontal dashed lines indicate the fold-change and *p*-value cutoffs; (**B**): Relative effect sizes are plotted for significant taxa. Values >0 (red colors) indicate taxa that were elevated in relative abundance in the hypoxia group compared to controls. Values <0 (blue colors) indicate taxa that were enriched in the control group compared to hypoxia; (**C**): Alpha-diversity differs between hypoxia and control groups. Boxplots summarize distributions of three measures of alpha-diversity between the Co and Hx groups. *p*-values were ascertained by Student’s t-test with the Welch approximation. Sobs: Species observed (number of taxa observed in each sample). Hmax: maximum value of the Shannon Diversity index for each sample.

## Data Availability

The data presented in this study are available on request from the corresponding author.

## Appendix A

### Appendix A.1. Chronic Hypoxia-Induced Intestinal Dysbiosis and Decreased Microbial Diversity in SD Rats

To examine if PH is characterized by alterations of gut microbiota, we used 16S rRNA gene sequencing to profile fecal bacteria in control and chronically hypoxic SD rats. Hypoxia had minimal effects on overall microbiota composition (i.e., beta-diversity; *p* = 0.083). Nonetheless, multiple taxa differed in relative abundance between control and hypoxic groups (Figure A1A,B), using a nominal *p*-value cutoff of 0.05 (only *Allobaculum* and *Coprococcus* had FDR-corrected *p*-values < 0.05). Genera belonging to the phyla Firmicutes (*Allobaculum*), Actinobacteria (*Bifidobacterium*, *Enterorhabdus*), and Tenericutes (*Anaeroplasma*), and Proteobacteria (*Parasutterella*) phylum were more abundant in control animals. In contrast, several bacteria phyla Firmicutes (*Coprococcus*, *Oscillinacter*, *Anaerotruncus*, *Papillibacter*, *Blautia*) and Bacteroidetes (*Prevotellaceae*) were upregulated in chronically hypoxic hypertensive animals, which is consistent with a previous report showing increased Firmicutes in hypertension. Furthermore, alpha-diversity (Figure A1C) was lower in hypoxic animals as measured by evenness (*p* = 0.083) and Shannon diversity (*p* = 0.044) indices.

### Appendix A.2. Microbiome Data Analysis

Fecal samples of control and chronically hypoxic hypertensive animals, either treated or untreated with butyrate, were subjected to 16S rRNA gene profiling following our standard procedures [89]. PCR products were normalized using a SequalPrep^TM^ kit (Invitrogen, Carlsbad, CA) and paired-end sequencing was performed on the Illumina MiSeq platform using a 600-cycle version 3 reagent kit. The 16S rRNA gene sequences were demultiplexed, quality filtered, culled of human and chimeric sequences [90], and classified using the SINA/SILVA platform as previously described [91]. Operational taxonomic units (OTUs) were produced by clustering sequences with identical taxonomic assignments. A median of 98,216 (minimum 57,973, maximum 132,957) reads was generated per sample and Good’s coverage was >99.0% for all samples, indicating an excellent depth of sequence coverage. Relative abundances (RA) were calculated as the number of sequences for a specific taxon standardized to the total number of sequences in that sample. Common measures of biodiversity (Good’s coverage, S_obs_, Shannon diversity H, Shannon evenness (H/H_max_)) were estimated through 1000 re-samplings at a rarefaction point of 50,000 sequences with the Explicet package [92]. For microbiome analysis, differences in overall composition (i.e., beta-diversity) were assessed through permutational ANOVA (PERMANOVA) using the Bray–Curtis dissimilarity index. PERMANOVA *p*-values were inferred through 10^6^ label permutations. Individual taxa differing between treatment groups were identified using the ANOVA-like differential expression (ALDEx2) R package [93], which takes into account the compositional nature of microbiome datasets. Alpha-diversity indices (i.e., S_obs_, Shannon H, Shannon H/Hmax) were assessed by unpaired *t*-test with Welch’s correction.
